# Cigarette Smoke-Induced Acquired Dysfunction of Cystic Fibrosis Transmembrane Conductance Regulator in the Pathogenesis of Chronic Obstructive Pulmonary Disease

**DOI:** 10.1155/2018/6567578

**Published:** 2018-04-23

**Authors:** Juan Shi, Hui Li, Chao Yuan, Meihui Luo, Jun Wei, Xiaoming Liu

**Affiliations:** ^1^College of Clinical Medicine, Ningxia Medical University, Yinchuan, Ningxia 750004, China; ^2^Ningxia Key Laboratory of Clinical and Pathological Microbiology, General Hospital of Ningxia Medical University, Yinchuan, Ningxia 750004, China; ^3^Ningxia Institute for Stem Cell Research, General Hospital of Ningxia Medical University, Yinchuan, Ningxia 750004, China; ^4^College of Life Science, Ningxia University, Yinchuan, Ningxia 750021, China

## Abstract

Chronic obstructive pulmonary disease (COPD) is a disease state characterized by airflow limitation that is not fully reversible. Cigarette smoke and oxidative stress are main etiological risks in COPD. Interestingly, recent studies suggest a considerable overlap between chronic bronchitis (CB) phenotypic COPD and cystic fibrosis (CF), a common fatal hereditary lung disease caused by genetic mutations of the cystic fibrosis transmembrane conductance regulator (CFTR) gene. Phenotypically, CF and COPD are associated with an impaired mucociliary clearance and mucus hypersecretion, although they are two distinct entities of unrelated origin. Mechanistically, the cigarette smoke-increased oxidative stress-induced CFTR dysfunction is implicated in COPD. This underscores CFTR in understanding and improving therapies for COPD by altering CFTR function with antioxidant agents and CFTR modulators as a great promising strategy for COPD treatments. Indeed, treatments that restore CFTR function, including mucolytic therapy, antioxidant ROS scavenger, CFTR stimulator (roflumilast), and CFTR potentiator (ivacaftor), have been tested in COPD. This review article is aimed at summarizing the molecular, cellular, and clinical evidence of oxidative stress, particularly the cigarette smoke-increased oxidative stress-impaired CFTR function, as well as signaling pathways of CFTR involved in the pathogenesis of COPD, with a highlight on the therapeutic potential of targeting CFTR for COPD treatment.

## 1. Introduction

Chronic obstructive pulmonary disease (COPD) is one of the most prevalent causes of mortality in the aging population worldwide, which is characterized by an irreversible chronic airflow limitation [1]. Emphysema and chronic bronchitis (CB) are two major clinical and epidemiological phenotypes of this chronic lung disease. Pathologically, inflammation in small airways (CB) and destruction of lung parenchyma (emphysema) are hallmarks of COPD. Of note, most patients with COPD exhibit symptoms of both CB and emphysema [2], although CB is typically the one predominant clinical phenotype in COPD patients [3]. Etiologically, cigarette smoke (CS) and the CS-caused oxidative stress are considered as the most common etiological factors in COPD. Clinically, a subgroup of patients with emphysema phenotype of COPD also develops CB accompanied by inflammatory airway wall thickening and/or bronchiectasis [4–6]. These manifestations support that the airway mucus obstruction may be a crucial factor in the pathogenesis of chronic inflammation driving disease progression in COPD [7, 8].

Cystic fibrosis (CF) is a fatal heterogeneous recessive genetic disorder caused by mutations in the cystic fibrosis transmembrane conductance regulator (CFTR) gene, which is characterized by chronic bacterial infection in airways and sinuses, pancreatic exocrine insufficiency, and elevated concentrations of chloride in sweat [9]. Clinicopathologically, CF is a multiple organ disorder including airways and lung, pancreas, gastrointestinal tract, and reproductive organs. However, the disorder in airways and lung is considered as a primary cause of morbidity and is responsible for 85% of deaths in CF patients. It is therefore also a model of obstructive lung disease [10].

CFTR is an adenine nucleotide-binding cassette (ABC) protein and anion channel [11], which is responsible for the transportation of Cl^−^ and HCO_3_^−^ anions into the airway lumen, along with Na^+^ and H_2_O following passively through the paracellular pathway, resulting in an isotonic increase in height/volume of airway surface liquid (ASL) [12]. Mutations in the CFTR gene lead to the dysfunction or deficiency of the CFTR protein, which in turn results in a decreased ASL volume and subsequent mucus dehydration/stasis, which in turn impair mucus clearance and the lung's innate defense [13, 14].

**Figure 1:**
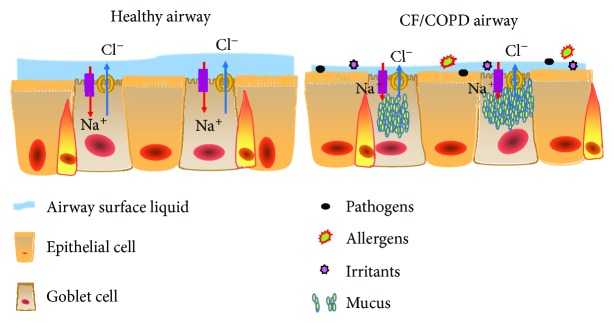
Model of airway surface dehydration (mucus hyperconcentration) in chronic obstructive pulmonary disease (COPD). The healthy airway surface (left panel) is covered with a thin film of mucus able to entrap inhaled insults that are constantly removed from the lungs by mucociliary clearance. The proper function of this innate airway defense mechanism largely relies on the function of CFTR, ENaC, and other alternative Cl^−^ channels. In the COPD airway (right panel), the dysfunction of CFTR-mediated chloride channel leads net absorption of sodium leads to dehydration of airway surfaces, decreases ASL volume, and impairs mucus stasis and clearance.

Interestingly, airway mucus obstruction is an important hallmark of both CF and COPD, particularly in the CB form of COPD. For example, the clinical manifestations of CB include sputum production and impaired mucus clearance with chronic inflammation, which are similar to clinical features in early CF lung disease [13]. Furthermore, studies on the CB phenotype of COPD have identified that the impaired mucociliary clearance (MCC) of airways is a critical pathological process that drives disease initiation and progression. Mechanistically, the efficiency of MCC is largely dependent on functions of CFTR and epithelial Na^+^ channel (ENaC), ciliary beating, and appropriate rates of mucin secretion [15]. Even more importantly, the oxidative stress and toxic components of cigarette smoking (CS) are major causes of COPD, which can also lead to a decrease in the cellular levels of CFTR in airway epithelia [16]. Indeed, a compelling body of evidence supports the above findings; that is, the chronic CS exposure may induce the acquired CFTR dysfunction, subsequently contributing to the pathogenesis of CB [17–26]. Therefore, the pathophysiology of CF and COPD has been proposed to share a similar process of initiation and progression, including the decrease of the height/volume of ASL and mucus dehydration/stasis/accumulation (Figure 1). Molecularly, the CFTR protein dysfunction is commonly seen in both disease conditions, except that the CFTR dysfunction is caused by genetic mutations in CF, while an acquired CFTR dysfunction caused by CS and oxidants is defined in the CB form of COPD [13]. These studies thus further highlight that lessons learned from CF may be applicable to COPD, suggesting that current therapeutic strategies in CF may be translated to COPD treatment, particularly in the CB phenotype of COPD. In this article, we summarize the implication of CFTR dysfunction that is induced by cigarette smoke exposure and oxidants in the development and progression of COPD, expound our current understanding of mechanisms of acquired CFTR dysfunction in the pathogenesis of COPD, and highlight the potential of recent breakthroughs in targeting CFTR in COPD treatment.

## 2. Cigarette Smoke-Increased Oxidative Stress Impairs the Expression and Function of CFTR

Although it has been well established that chronic cigarette smoke exposure is a principal etiological factor for COPD, mechanisms underpinning the pathogenesis of COPD remain largely unknown. Etiologically, a compelling body of evidence has demonstrated that cigarette smoke- (CS-) related oxidants and calcium conspire to impair the expression and function of CFTR in airway epithelia at various National Natural Science Foundation of Chinaextents, through a variety of molecular mechanisms at different levels, including the reduced expression of the CFTR transcript, diminished CFTR protein by accelerating protein degradation, and altered channel gating, subsequently leading to an acquired CFTR dysfunction [13, 16]. In this regard, the CS showed an acute effect on airway ion transport and CFTR activity. The CFTR-mediated Cl^−^ secretion could be significantly reduced following either an acute or a chronic cigarette smoke exposure *in vitro* and *in vivo*, resulting in a reduced channel gating, open probability, and mucus transport, as well as airway surface dehydration. Of interest, a chronic exposure of airway epithelial cells to cigarette smoke (CS) could lead to a ~75% rapid decrease of the channel gating and open probability and an up to 60% inhibition of CFTR-mediated Cl^−^ secretion *in vivo* (Figure 1) [13, 18–20, 23]. Animal studies also experimentally demonstrated that the CS could reduce CFTR levels in lipid rafts, and the reduced CFTR level was involved in epithelial cell apoptosis and autophagy induced by CS [27].

Indeed, the above studies implied that acquired CFTR dysfunctions, along with the presence of CS-induced goblet cell metaplasia and mucin hypersecretion led to the failure of maintaining the proper mucus hydration of ASL in smokers, thus increasing the risk of mucus stasis and chronic airway diseases [7, 28]. This notion was further supported by evidence that smokers with COPD exhibited a reduced CFTR-mediated Cl^−^ secretion in the upper and lower airways, and such a deficient ion transport was correlated with airway mucus dehydration [20]. Of great interest, the CS-induced CFTR dysfunction was also found in extrapulmonary disorders that are characterized in CF, such as pancreatitis, male infertility, and cachexia, suggesting that cigarette smoke could cause systemically impaired CFTR, probably via a circulating agent [22].

In addition, acrolein, ceramide, and cadmium are main three constituents of cigarette smoke metabolites, which have been implicated in CFTR dysfunctions *in vitro* and *in vivo* [22–24, 29]. Among them, acrolein is a highly reactive cigarette smoke metabolite that forms covalent adducts with DNA and proteins [30] and is able to block CFTR by directly inhibiting channel gating [22]. As a major component of cigarette smoke, cadmium is also a prevalent environmental contaminant. Exposure to cadmium was found to inhibit the expression of the CFTR protein and subsequent chloride transport in human airway epithelial cells *in vitro* and human COPD lungs [31] and lungs of mice that were intranasally instilled with cadmium *in vivo* [32].

Similarly, more recent studies demonstrated that the smoke-induced accumulation of ceramide was associated with the inhibition of CFTR expression and the severity of emphysema in COPD patients, in whom the CS-induced ceramide accumulation mediated the pathogenesis of COPD through a mechanism by activating NF-*κ*B signaling and inducing epithelial cell apoptosis [17, 27, 33]. In order to explore how membrane CFTR modulated ceramide signaling in lung injury and emphysema, Bodas et al. evaluated CFTR in CFTR-deficient mice and COPD lung tissues [27, 33]. Their results revealed an inverse correlation between the expression of CFTR and severity of emphysema and ceramide accumulation in COPD patients compared with healthy subjects [33]. In addition, *Pseudomonas aeruginosa*LPS-induced lung injury could be much more effectively controlled by chemically inhibiting the de novo ceramide synthesis in CFTR wild-type mice relative to CFTR-deficient mice [27]. Further studies demonstrated that the CFTR-dependent lipid rafts and ceramide signaling played a modulatory role in CS-induced lung epithelial injury and emphysema, indicating that membrane CFTR was essential for regulating lipid raft ceramide accumulation and inflammatory signaling [27, 33]. In addition to lung epithelial cells, CS could also alter CFTR lipid rafts in macrophages and impair macrophage bacterial phagocytosis and killing [34]. These studies clearly suggest that CS-induced acquired CFTR dysfunction contributes to the development and progression of COPD.

## 3. Implications of CFTR in COPD Pathogenesis

The CFTR-mediated chloride channel plays a pivotal role in vital cellular signaling processes contributing to cell homeostasis. A reduced or dysfunctional CFTR may lead to an impaired efflux of cell anions such as chloride and bicarbonate. A failure of anion transportation of epithelial cells may yield to an increased viscosity of secretions and mucus accumulation, impaired ASL homeostasis, reduced clearance of bacteria, and enhanced chronic infection and inflammation. Those ultimately promote the obstruction and fibrosis of epithelial tissues and organs, particularly of the lung [35]. Indeed, accumulating evidences have suggested the implication of CFTR dysfunction in the pathogenesis of COPD, which are listed in Table 1.

Owing to the similarities of several clinical manifestations between COPD and CF disease, the CFTR has been considered to be implicated in the pathogenesis of COPD. Genetically, the correlation between CFTR gene mutations and the risk of COPD was first examined. In this respect, a deletion of three nucleotides encoding phenylalanine at position 508 of the CFTR gene, delta F508 (ΔF508), is the most common CF-causing mutation. The ΔF508 mutation results in a 50% decrease in CFTR protein density and function in individuals carrying a heterozygous ΔF508 mutation compared to those carrying a wild-type CFTR gene [40]. Intriguingly, human studies failed to determine an increased risk for COPD in carriers of the CFTR ΔF508 mutation [41, 42]. Moreover, the degree of reduction in CFTR protein function in ΔF508 heterozygous tracheal epithelial cells exposed to CS was not different from that in wild-type CFTR cells *in vitro*, although ΔF508 carriers clinically exhibited a significant decrease in pulmonary functions of forced expiratory volume in one second (FEV1) and forced vital capacity (FVC) as compared with subjects bearing a non-CFTR mutation [43]. Unlike that seen in CFTR ΔF508 carriers, however, several early studies have identified an increased incidence of the CFTR R75Q and M470V mutation alleles in individuals with COPD [36, 44, 45]. In this regard, a 3.4-fold decrease in the risk of severe COPD was demonstrated in a Serbian population carrying the CFTR M470V variant genotype [46], while an increased risk of COPD was examined in subjects bearing a R75Q CFTR variation [45].

Of note, it has been well established that cigarette smoking-induced oxidative stress and/or inflammation can decrease CFTR activity and impair mucociliary transport in airway epithelial cells. But the presence of CFTR mutation heterozygosity had no impact on the CS-induced reduction of CFTR activity, which did not increase the risk of COPD with CB either [43]. On the other hand, a reduced CFTR activity in the small airway was determined by transepithelial potential differences in smokers with or without COPD in comparison with healthy nonsmokers, and the difference was statistically significantly associated with symptoms of dyspnea and CB [20], although healthy cigarette smokers bearing no CFTR gene mutations also exhibited a marked decrease in CFTR function as determined by nasal potential difference (NPD) measurements [19]. These studies clearly imply that acquired CFTR dysfunctions induced by cigarette smoking may contribute to the pathophysiology of COPD. Indeed, this notion was further experimentally supported by several studies in murine models [37, 47]. For instance, mice overexpressing the beta subunit of ENaC (*β*-ENaC) displayed a decreased CFTR function, along with an airway mucus obstruction and mortality, which were consistent with the role of CFTR in defining manifestations of the severity of COPD [37]. Furthermore, a sustained reduction of CFTR protein expression was found to correlate with the smoke-induced emphysema in mice with different genetic backgrounds (C57BL/6, ApoE2/2, A/J, CD1, and Nrf22/2) [47]. In humans, the abundance of CFTR protein in the lung tissue of patients with pulmonary emphysema was strikingly correlated with lung function (FEV1 and FVC) and inversely correlated with their COPD stages [33, 38].

Apart from the association of the loss of CFTR activity and manifestations in COPD lungs, a correlation between the dysfunction of systemic CFTR and abnormal gland secretion in skins has also been defined in COPD patients; that is, the *β*-adrenergic sweat rate was significantly reduced in COPD patients, which was associated with the severity and clinical symptoms of COPD [39, 48]. In addition, transcriptome meta-analysis using 13 independent microarray datasets from CF; chronic pulmonary disorders, including COPD, IPF, and asthma; and environmental conditions, such as smoking and epithelial injury, recently identified that the CFTR gene was of potential therapeutic significance for these disorders [49]. This study also confirmed remarkable similarities in gene expression profiles between CF and COPD [49]. Taken together, these studies strongly support the hypothesis that acquired systemic CFTR dysfunctions induced by smoking play a causative role in COPD pathogenesis and contribute to the onset and severity of COPD, suggesting that CFTR may serve as a biomarker and potent druggable target for COPD treatment.

## 4. Possible Mechanisms of Cigarette Smoke-Induced CFTR Dysfunction

As mentioned in previous studies, CS could alter CFTR function by modulating the transcriptional expression of the gene, stability of the transcript, and stability and function of the protein [50]. In addition, numerous components, metabolites, and oxidants of CS were also able to modulate distinct CFTR functions at any steps of gene expression, stability, trafficking, and modification of CFTR protein [13, 16]. Apart from CS, other environmental factors or developed agents, such as oxidative agents, are also able to modulate CFTR function in COPD (Table 2).

**Figure 2:**
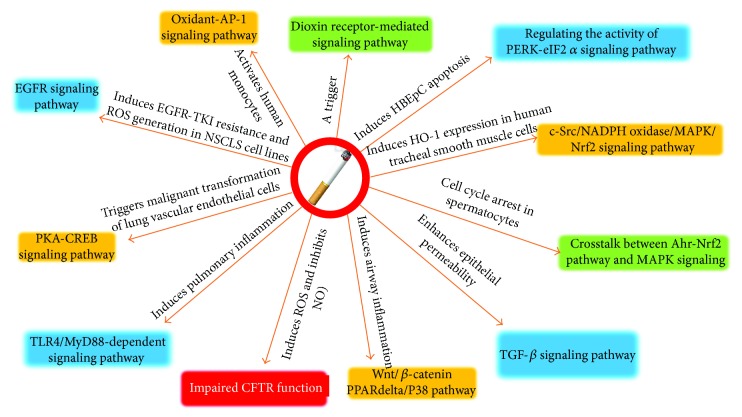
Signaling of oxidative stress activated by cigarette smoke. Cigarette smoke induces oxidative signaling and inflammatory responses. In this respect, cigarette smoke induces ROS production and impairs CFTR function, which is also a trigger of oxidative stress to activate the dioxin receptor-mediated signaling pathway and induce ROS production and cell cycle arrest or apoptosis and other signaling pathways.

In this regard, both gaseous and soluble phases of CS are sources of oxidants, as CS can highly oxidize [58]. Of interest, oxidative stress is a well-known inhibitor of CFTR gene expression and trigger of inflammation (Figure 2) [59]. Vice versa, the impaired or defective CFTR is able to lead an increased production of reactive oxygen species (ROS) by activation of NADPH oxidase (NOX/DUOX) family members [50, 60]. Therefore, oxidation alone has been suggested as one of the most important factors that had impacts on CFTR gene expression, protein density, and channel function [50, 60]. Therefore, chronic exposure of lung cells to cigarette metabolites of oxidants was found to result in endoplasmic reticulum stress, unfolded protein response, accumulated ceramide, and increased cell apoptosis [50]. For instance, the defective CFTR encoded by ΔF508 gene mutation could induce oxidative inflammatory stress by ROS activation, which in turn induced autophagy impairment and accumulation of CFTR in aggresome bodies [17].

In addition to the above direct effects of oxidative stresses of CS and its metabolites, such as acrolein, ceramide, and cadmium on CFTR function, several other potential mechanisms have also been proposed to account for CFTR dysfunctions. One additional mechanism is that CS was able to impair CFTR trafficking by inducing protein internalization attributed by an acute misfolding of surface CFTR, which could rapidly clear CFTR from the plasma membrane [13, 61]. Intriguingly, the cigarette smoke-induced internalization of CFTR was not colocalized with lysosomal proteins but with the intermediate filament vimentin, indicating that the CFTR was trafficked into an aggresome-like perinuclear compartment of cells [13]. This was similar to that seen in cells expressing ΔF508 CFTR [17]. Mechanistically, the CS could induce Ca^2+^ release from lysosomes and increase cytoplasmic Ca^2+^ concentration, which in turn inhibits the normal route of CFTR sorting/degradation and reroutes CFTR internalization to aggresomes [16, 62]. This hypothesis was supported by an evidence that macrolide antibiotic bafilomycin A1 could inhibit the smoke-induced Ca^2+^ release and prevent CFTR internalization and clearance from the plasma membrane [62].

Together with the fact that chronic oxidative insults including CS, hypoxia, and chronic inflammation are the causes in the majority of COPD cases and oxidative stresses are able to impair CFTR function and are implicated in the pathogenesis of COPD, these studies suggest a common mechanism between COPD and CF disease (Figure 2). Therefore, a strategy restoring CFTR function may offer an opportunity for the treatment of COPD.

## 5. CFTR as a Potential Therapeutic Target for COPD

COPD has emerged as one of the most prevalent chronic diseases in the aging population and has become the fourth leading cause of death [63]. Unfortunately, there is no pharmacologic treatment currently available to alter the progressive decline in lung functions that ultimately leads to the disability and death of COPD patients [64]. Therefore, there is an ongoing unmet need to develop novel and effective agents for COPD treatments. Fortunately, owing to the overlap between COPD and CF in several key manifestations, including mucus hypersecretion, reduced mucociliary clearance, small airways' mucus obstruction, chronic bacterial infections and airway inflammation, and goblet cell metaplasia [65], together with the pathophysiological link of impaired CFTR function between these two chronic pulmonary diseases, therapeutic strategies for CF disease and pharmaceutical agents that target CFTR may therefore offer therapeutic effects in COPD patients.

**Figure 3:**
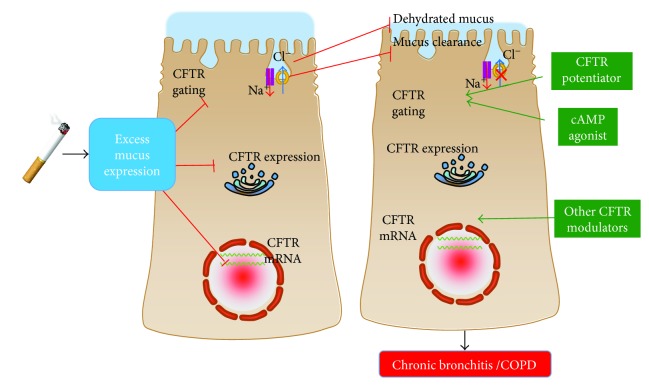
The cystic fibrosis transmembrane conductance regulator (CFTR) is a potential target for COPD treatment. The proper CFTR function is critical in maintaining the homeostasis of airway surface hydration and mucociliary clearance of normal airway epithelia. Cigarette smoke is able to induce excessive mucus secretion and has a negative impact on CFTR activation. The consequences of genetic and acquired CFTR dysfunction in patients with CB and COPD lead to a disreputable homeostasis of mucus and decreased ASL volume. The dehydrated mucus impairs the mucus clearance. Therefore, strategies that restore the CFTR function at different levels (mRNA and protein expression, stability, CFTR gating, and trafficking) using CFTR modulators (potentiator/corrector) and cAMP agonist (PDE inhibitors) may provide novel therapeutic approaches in obstructive pulmonary diseases, such as CB of COPD. The image on the left panel shows the cigarette smoke-induced CFTR dysfunction at different levels, and that in right the panel shows potential therapeutic interventions to restore CFTR function in cigarette smoke-exposed CFTR dysfunction. The red blocked line indicates an inhibition, and the green line with arrow represents an induction.

Accordingly, this concept has motivated researchers to investigate CFTR dysfunction as a possible joint therapeutic target for CF, COPD, and other chronic airway diseases. There are indeed several therapeutic strategies, and approved and underdeveloped agents targeting CFTR channels have been attempted or translated into new treatments for mucus obstructive pulmonary diseases that share the same pathophysiology with CF, such as COPD, among which antioxidant ROS scavenger, mucus rehydration and mucolytic therapy, CFTR stimulator (phosphodiesterase (PDE) inhibitor), and CFTR potentiators gain the most interest in testing for COPD treatments (Figure 3).

Since the CB form of COPD is a disease at least in part related to CFTR dysfunction and mucus dehydration, the ASL/mucus rehydration may be thus beneficial in airway obstructive mucus clearance in COPD patients. Several strategies including osmotic agent-mediated direct rehydration, ENaC inhibition, and CFTR function restoration have been used for the increase of ASL volume/mucus rehydration [13, 66]. For example, the aerosol delivery of hypertonic saline exhibited a promise by restoring rehydration, increasing ASL volume, improving mucus clearance and lung function, and accelerating mucociliary clearance (MCC) in CF and COPD patients, with an exception that the duration of action was relatively short in normal and COPD airways in comparison with CF airways [13, 66]. Mechanistically, this scenario likely reflects the fact that the hypertonic saline results in a high salt concentration on airway surfaces, which in turn generates a gradient of Cl^−^ to be absorbed through the partially functional CFTR-mediated channel and the paracellular pathway, together with the absorption of Na^+^ by ENaC and the paracellular pathway, and these thus may limit the sustainability of osmotic effects on the surfaces of COPD normal airways. Conversely, the fully defective CFTR function in CF airways completely loses the CFTR-mediated transcellular path for Cl^−^ absorption, limiting the Na^+^ absorption and yielding the sustenance of the high concentration of NaCl on airway surfaces [13]. In addition, a preclinical study interrogating the hydration strategies in COPD mouse model using hypertonic saline and preventive inhibition of the amiloride-sensitive epithelial Na^+^ channel also demonstrated the effectiveness of hydration strategy in unplugging airway COPD, suggesting that hydration agents may be a promising therapeutic strategy to unplug mucus in the CB form of COPD [66], although currently such a mucolytic therapy only showed marginal benefits in COPD patients [67, 68].

CS is an important source of oxidative stress. Its induced intracellular ROS is a causative factor of COPD and a well-known CFTR inhibitor; therefore, several ROS scavengers have been expected to partially restore CFTR function and be used for COPD treatment. For instance, cysteamine is a reduced form of cystamine, which is an approved agent with antioxidant, antibacterial, and mucolytic properties. This agent has been shown to decrease lung inflammation and improve lung function in CF patients by potentially restoring autophagy and allowing CFTR to be trafficked to the cell membrane in a recent clinical trial [17, 69]. In this context, accumulated polyubiquitinated proteins and impaired autophagy marker p62 of aggresome bodies were determined in cigarette smoke-exposed lung epithelial BEAS2 cells and murine lungs. In addition, increased aggresome bodies were also found in the lungs of smokers and COPD patients, which were correlated with the severity of emphysema and alveolar senescence, suggesting that cysteamine was capable of modulating the cigarette-induced pathogenesis of the emphysema phenotype of COPD by restoring impaired autophagy and CFTR function [17, 69, 70].

Nitric oxide (NO) and S-nitrosoglutathione (GSNO), members of the S-nitrosothiol (SNO) group of bioactive NO reservoirs, play a crucial role in maintaining lung functional homeostasis under a physiological condition, in which intracellular levels of GSNO are controlled by the enzyme of S-nitrosoglutathione reductase (GSNOR) that degrades GSNO [71, 72]. Intriguingly, a recent study revealed low NO levels, along with an increased level of serum asymmetric dimethylarginine (ADMA) in COPD patients and current smokers, which was correlated with the severity of COPD, highlighting the importance of NO signaling in COPD emphysema pathogenesis [73, 74]. Equally noteworthy, a reduced GSNO level was determined in CF patients, consisting of the finding that GSNO played important roles in modulating the expression, maturation, and function of CFTR protein [75, 76]. Therefore, increasing GSNO levels may be a promising strategy for treating obstructive lung disease, owing to the ability of GSNO to increase the maturation/expression of CFTR by facilitating its rescue from aggresome bodies [71, 76]. Indeed, therapeutic benefits of augmentation of GSNO or treatment with GSNOR inhibitor (N6022) have been tested in several clinical trials.

A recent study on the effects and mechanism of GSNO augmentation in regulating inflammatory oxidative stress and COPD emphysema pathogenesis demonstrated that CFTR-colocalized aggresome bodies were correlated with an increasing emphysema severity in the lung of COPD subjects, and the treatment of GSNO or GSNOR inhibitor (N6022) could significantly inhibit cigarette smoke extract (CSE)-induced decrease of membrane CFTR, through a mechanism that involves rescuing CFTR from ubiquitin (Ub)-positive aggresome bodies and inhibiting CFTR protein misfolding. In addition, GSNO restoration could significantly inhibit CSE-induced ROS activation, chronic hypoxia- (Ch-) CS-induced perinuclear CFTR protein accumulation, and autophagy impairment [17]. These studies thus suggested that increasing GSNO levels could prevent the pathogenesis of COPD emphysema by reducing CS-induced acquired CFTR dysfunction [17].

Since CFTR dysfunction can lead to a reduced responsiveness to the cyclic adenosine monophosphate (cAMP)/protein kinase A (PKA) signaling pathway, pharmacological agents able to elevate intracellular cAMP have been used for the treatment of CF disease. In this context, phosphodiesterases (PDEs) can break down cAMP to regulate intracellular cAMP concentrations and diffusion; therefore, PDE inhibitors are able to inhibit cAMP breakdown and restore CFTR function. In this regard, selective PDE4 and dual PDE3/4 inhibitors have anti-inflammatory and bifunctional bronchodilator effects in a preclinical study [77]. In this regard, the dual PDE3/4 inhibitor RPL554 has exhibited an ability to activate CFTR-dependent ion secretion in human primary bronchial epithelial cultures of CF patient carrying the R117H/F508del mutations [78]. To date, however, only one selective PDE4 inhibitor, the orally active roflumilast, has been licensed for the treatment of CB form of COPD [79].

Roflumilast was originally used for the treatment of inflammation in COPD. It could activate CFTR-dependent Cl^−^ secretion by a cAMP-mediated pathway [40]. Since PDE4 is predominant in airway epithelial cells, roflumilast can induce or activate CFTR by increasing cAMP. Indeed, its ability to partially restore the CS-induced CFTR dysfunction in human bronchial epithelial cells (HBECs) [55] and increase CFTR mRNA levels in cell cultures exposed to cigarette smoke [54] has been shown. Such a roflumilast-restored CFTR-mediated chloride transport was further validated in CS-exposed mice [29]. In this study, A/J mice were exposed to CS or air for induction of CFTR dysfunction before they were treated with roflumilast. The NPD *in vivo* and short-circuit current (Isc) analysis of trachea ex vivo were used for accessing CFTR-dependent chloride transport. Of note, the acute roflumilast treatment led to an increased CFTR-dependent chloride transport in mice exposed to smoke or air. Moreover, the smoke-impaired CFTR function of mice could be completely reversed after oral administration of roflumilast for five weeks, further implicating the CFTR activation as a mechanism by which COPD patients with CB benefits from roflumilast treatment [29].

In addition to COPD treatments with PDE inhibitors by activating wild-type CFTR, a direct stimulation of CFTR activity with CFTR modulator may offer an alternative means to restore CFTR function in COPD [26, 80]. In this respect, CFTR potentiators are a class of agents able to correct gating defects of mutant CFTR [81, 82]. Among them, ivacaftor (formerly VX-770) is a FDA-approved agent for the treatment of CF with 33 different CFTR mutations [23, 83]. Although CFTR potentiators were originally developed for restoring the function of mutant CFTR, some of them including ivacaftor were also able to enhance wild-type CFTR activity by augmenting open channel probability [25, 84]. Indeed, human bronchial epithelial cells (HBECs) coexposed to ivacaftor and cigarette smoke extract (CSE) showed an increased CFTR-mediated Cl^−^ secretion relative to cells exposed to CSE alone [23, 25].

Discouragingly, a recent pilot study evaluating ivacaftor treatment for 12 current or former smokers with COPD and CB, however, reported that there was no significant improvement in CFTR function in patients receiving ivacaftor compared to those receiving placebo, as ascertained by improving sweat chloride concentrations and NPD [85]. In addition, a nonsignificant improvement in symptoms was observed in ivacaftor-treated patients either, as accessed by changes in the breathlessness, cough, and sputum scale (BCSS). Of interest, patients with the highest sweat chloride concentration at baseline also had the largest improvement in sweat chloride and BCSS, implying that patients with more severe CFTR dysfunction might benefit more from ivacaftor treatment at some extent by partially reversing CFTR function [85]. Conversely, chronic exposure of ivacaftor was found to inhibit wild-type CFTR by shortening the dwell time of CFTR in the plasma membrane, indicating that ivacaftor may not be the potentiator to reverse CFTR dysfunction in COPD patients [86]. However, improvements in CFTR activity and respiratory symptoms were observed in severe COPD patients receiving ivacaftor. This suggests that it is of importance to identify the most appropriate patient phenotypes involving differentiation based on the level of concomitant emphysema, bronchiectasis and disease severity, the status of smoking, and baseline CFTR function (and/or CFTR genotype) [24, 26].

## 6. Conclusions

Obstructive airway diseases, especially COPD, are common chronic diseases causing morbidity and mortality in the aging population worldwide. No pharmacotherapy is currently available for COPD; thus, there is an unmet need for novel and effective therapies to combat this disorder. Mechanistically, a compelling body of study has suggested that the acquired CFTR dysfunction induced by CS and its increased oxidative stress at least in part plays a pathogenic role in obstructive airway diseases other than the CF, such as COPD, nonatopic asthma, and non-CF bronchiectasis. In this regard, even a small decrement in CFTR function may likely impose a striking effect on airway physiology and mucus clearance. In the case of COPD, CS-induced CFTR dysfunction and decrease of anion transport have largely been validated. In this context, toxic agents within CS are able to alter CFTR gene expression, protein stability, and anion conductance in healthy smokers and in COPD subjects, representing a novel target of CFTR for the development of therapeutic strategies. Therefore, targeting CFTR has recently spurred an increased interest in the treatment of CB phenotype of COPD [80].

Indeed, therapeutic strategies that reverse CFTR functions, including mucolytic therapy, antioxidant ROS scavenger, CFTR stimulator (PDE inhibitor), and CFTR potentiator, are under testing for COPD treatments (Figure 3). More importantly, several newly approved and developed CFTR modulators have offered a novel treatment opportunity for COPD. The introduction of both cAMP-dependent CFTR modulator (PDE inhibitor: roflumilast) and cAMP-independent CFTR modulator (CFTR potentiator: ivacaftor) has shown promise in potentiating CFTR function with increased CFTR-mediated Cl^−^ ion transportation in the treatment of acquired CFTR dysfunction in COPD. Particularly, the FDA's newly approved CFTR potentiator ivacaftor has been recently tested for safety and early efficacy in patients with the CB form of COPD. Together with the increasing evidence for therapeutic effects of CFTR restoration in COPD, it is reasonable to expand the investigation of CFTR modulators to this disorder.

In addition to roflumilast and ivacaftor, the next generation of potentiators targeting the CFTR gating defect in smoke-exposed human tissues and CFTR correctors (VX809, lumacaftor, and ABBV/GLPG-2222) restoring and/or enhancing CFTR trafficking to cell surface may provide novel strategies in COPD treatments [87]. Moreover, the combination of CFTR potentiator with CFTR corrector and/or PDE inhibitor may synergistically increase the effect of the restoration of CFTR function [78, 88]. For example, the CFTR potentiator VX-770 (ivacaftor) was found to further enhance dual PDE3/4 inhibitor RPL554-induced CFTR activity in primary HBECs [78]. Of note, the therapeutic strategies that restore CFTR function using PDE inhibitors and CFTR potentiators/correctors are relatively new; therefore, developing novel compounds or approaches to reverse CFTR dysfunction in cigarette smoke-exposed/CB airways may provide effective treatments for COPD.

## Figures and Tables

**Table 1:** Studies on implications of CFTR in COPD.

Object of study	Model	Molecular mechanism	Effect	Ref
Correlation of CFTR mutations and COPD	Subjects including 20 patients with asthma, 19 with DB, and 12 with COPD	CFTR gene mutations	The hyperactive M470 allele was more frequent in COPD patients	[36]
Impact of ENaC on CFTR function	C57BL/6 and BALB/c mice overexpressing the beta-ENaC subunit	Overexpressing ENaC impaired CFTR function	Dysfunction of CFTR contributed to the onset and severity of COPD	[37]
Impact of cigarette smoke on CFTR function	Current and former smokers with or without COPD	Cigarette smoke induced CFTR dysfunction and correlated with COPD disease phenotype	Cigarette smoke induced the acquired CFTR dysfunction and contributes to COPD pathogenesis	[20]
CFTR in COPD pathogenesis	GOLD 0/4 patients and HBECs	Cigarette smoke reduced the expression of CFTR protein and reduced airway surface liquid height	Cigarette smoke induced CFTR dysfunction and correlated with COPD disease phenotype	[31]
ENaC and CFTR in COPD pathogenesis	ATI cells and ATII cells in distal lung tissues	Augmentation of ENaC induced CFTR dysfunction and impaired lung function	ENaC and CFTR-mediated chloride channel are biomarkers and potent druggable targets of COPD	[38]
CFTR-mediated chloride channel in COPD	Healthy and COPD smokers	CFTR-mediated chloride channel detected by *β*-adrenergic sweat rate	CFTR-mediated chloride channel was significantly reduced in COPD smokers as detected by *β*-adrenergic sweat rate, which was associated with COPD severity and clinical symptoms of COPD	[39]
Cigarette smoke-impaired CFTR function	Cigarette smokers and patients with COPD	Cigarette smoke induced CFTR dysfunction by reducing CFTR mRNA, accelerating degradation, and altering channel gating	Acquired CFTR induced by cigarette smoke contributed to COPD with a clinical phenotype similar to mild CF	[23]

ATI: alveolar type I; ATII: alveolar type II; COPD: chronic obstructive pulmonary disease; CFTR: cystic fibrosis transmembrane conductance regulator; DB: disseminated bronchiectasis; ENaC: epithelial sodium channel.

**Table 2:** Impacts of cigarette smoke and other agents on CFTR function in COPD.

Agents	Model	Molecular mechanism	Effect	Ref
Cigarette smoke (CS)	Baby hamster kidney (BHK) cells	CS induced CFTR internalization and insolubility	CS-induced CFTR dysfunction led to airway surface liquid dehydration	[13]
	Endobronchial biopsy specimens	CS reduced lower airway CFTR activity in COPD patients	CS induced the acquired CFTR dysfunction contributing to COPD pathogenesis	[20]
	HBECs	Induced CFTR dysfunction	ENaC inhibition partially restored CFTR function and mucus hydration in CB patients	[51]
Cigarette smoke condensate (CSC)	Primary MNSE and HSNE	CSC affects the calcium-activated Cl^−^ transport pathway	CSC impaired CFTR functions in epithelial cells	[52]
Cigarette smoke extract (CSE)	Primary HBECs	Cigarette smoking transmits acute reductions in CFTR	CFTR potentiator (VX-770) reversed CFTR function	[23]
Tobacco carcinogen NNK transporter MRP2	Lung epithelial cells	Induced dysfunction of CFTR, MRP2, and PDZ proteins	Contributed to cigarette smoke-associated lung diseases, such as COPD and lung cancer	[53]
Roflumilast	Primary HBECs, Calu-3, and T84 monolayers	Roflumilast activated CFTR-mediated anion transport in airway and intestinal epithelia via a cAMP-dependent pathway	Roflumilast partially reversed the CS-impaired CFTR function and resulted in augmented ASL depth	[40]
	Primary NHBE and Vero cell VC-10	Roflumilast increased CFTR mRNA levels in CS-exposed cell cultures	Roflumilast can rescue smoke-induced mucociliary dysfunction by reversing decreased CFTR activity	[54]
	HBECs	Roflumilast restored CFTR function in CS-exposed cells	Roflumilast combined with adenosine increased mucosal hydration in HBECs exposed to CS	[55]
2-Cyano-3,12-dioxooleana-1,9-dien-28-oic acid (CDDO)	Human lungs	Chronic exposure of CS led to endoplasmic reticulum stress, unfolded CFTR protein response, and cell apoptosis	CDDO corrected defective Nrf2-dependent cellular response in chronic exposure of CS-induced lung disease	[56]
miR-101 and miR-144	HBECs and human lung tissues	Chronic exposure to CS upregulated miR-101 and miR-144, which suppressed CFTR in COPD lungs	miR-101 and miR-144 regulate the expression of the CFTR chloride channel in the lung	[57]
Ivacaftor, VX-770	Primary HBECs	Cigarette smoking transmitted acute reductions in CFTR activity due to inhibition of CFTR-dependent fluid transport	Cigarette smoke-reduced mucus transport in smokers could be reversed by CFTR potentiator VX-770	[23, 25]
Acrolein	Primary HBECs and A/J mice	Acrolein blocked CFTR by inhibiting channel gating	Acrolein mediated systemic CFTR dysfunction in smokers	[20]
Cadmium and manganese	16HBE14o-cells and human COPD lung tissues	Cadmium and manganese of CS reduced levels of CFTR protein and mRNA	Accumulation of cadmium and manganese reduced CFTR expression in the lungs of patients with severe COPD	[31]
S-Nitrosoglutathione (GSNO)	The preclinical COPD-emphysemamurine model	Alleviated CS-induced acquired CFTR dysfunction, resulting in autophagy impairment	Increasing GSNO levels reduced CS-induced acquired CFTR dysfunction and controlled COPD emphysema pathogenesis	[17]

ASL: airway surface liquid; BHK: baby hamster kidney; CB: chronic bronchitis; CDDO: 2-cyano-3,12-dioxooleana-1,9-dien-28-oic acid; ENaC: epithelial sodium channel; CS: cigarette smoke; CSC: cigarette smoke condensate; CSE: cigarette smoke extract; GSNO: S-nitrosoglutathione; HBECs: human bronchial epithelial cultures; HSNE: human sinonasal epithelial; MNSE: primary murine nasal septal epithelial; MRP2: multidrug resistance protein-2; NNK: 4-(methylnitrosamino)-1-(3-pyridyl)-1-buta-none; NHBECs: normal human bronchial epithelial cells.

## Abbreviations

AAT: Alpha-1 antitrypsin
ABC: Adenine nucleotide-binding cassette
ADMA: Asymmetric dimethylarginine
ASL: Airway surface liquid
ATI: Alveolar epithelial type I
ATII: Alveolar epithelial type II
BCSS: Breathlessness, cough, and sputum scale
cAMP: Cyclic adenosine monophosphate
CB: Chronic bronchitis
CD: Methyl-beta-cyclodextran
CDDO: 2-Cyano-3,12-dioxooleana-1,9-dien-28-oic acid
CF: Cystic fibrosis
CFTR: Cystic fibrosis transmembrane conductance regulator
Ch: Chronic hypoxia
COPD: Chronic obstructive pulmonary disease
CS: Cigarette smoke
CSC: Cigarette smoke condensate
CSE: Cigarette smoke extract
DB: Disseminated bronchiectasis
ΔF508: Delta F508
DUOX: Dual oxidase
ENaC: Epithelial Na^+^ channel
FEV1: Forced expiratory volume in one second
FHS/SHS: First-/second-hand cigarette smoke
FVC: Forced vital capacity
GOLD: Global Initiative for Chronic Obstructive Lung Disease
GSNO: S-Nitrosoglutathione
GSNOR: S-Nitrosoglutathione reductase
HBECs: Human bronchial epithelial cells
HSNE: Human sinonasal epithelia
IPF: Idiopathic pulmonary fibrosis
Isc: Short-circuit current
MCC: Mucociliary clearance
MNSE: Primary murine nasal septal epithelia
MRP2: Multidrug resistance protein-2
NNK: 4-(Methylnitrosamino)-1-(3-pyridyl)-1-buta-none
NO: Nitric oxide
NOX: NADPH oxidases
PDEs: Phosphodiesterases
PKA: Protein kinase A
RLMECs: Primary rat lung microvascular cells
ROS: Reactive oxygen species
S1P: Transport of sphingosine-1 phosphate.
